# Aligned copper nanorod arrays for highly efficient generation of intense ultra-broadband THz pulses

**DOI:** 10.1038/srep40058

**Published:** 2017-01-10

**Authors:** S. Mondal, Q. Wei, W. J. Ding, H. A. Hafez, M. A. Fareed, A. Laramée, X. Ropagnol, G. Zhang, S. Sun, Z. M. Sheng, J. Zhang, T. Ozaki

**Affiliations:** 1 Institut national de la recherche scientifique – Centre Energie, Matériaux et Télécommunications (INRS-EMT), 1650 Lionel-Boulet, Varennes, Québec J3X 1S2, Canada; 2 A*STAR Institute of High Performance Computing, Singapore 138632; 3 Max Planck Institute for Polymer Research, Ackermannweg 10, 55128 Mainz, Germany; 4 Physics Department, Faculty of Science, Helwan University, 11792, Cairo, Egypt; 5 SUPA, Department of Physics, University of Strathclyde, Glasgow G4 0NG, UK; 6 Laboratory for Laser Plasmas and Department of Physics and Astronomy, Shanghai Jiao Tong University, Shanghai 200240, China; 7 Collaborative Innovation Center of IFSA, Shanghai Jiao Tong University, Shanghai 200240, China

## Abstract

We demonstrate an intense broadband terahertz (THz) source based on the interaction of relativistic-intensity femtosecond lasers with aligned copper nanorod array targets. For copper nanorod targets with a length of 5 *μ*m, a maximum 13.8 times enhancement in the THz pulse energy (in ≤20 THz spectral range) is measured as compared to that with a thick plane copper target under the same laser conditions. A further increase in the nanorod length leads to a decrease in the THz pulse energy at medium frequencies (≤20 THz) and increase of the electromagnetic pulse energy in the high-frequency range (from 20–200 THz). For the latter, we measure a maximum energy enhancement of 28 times for the nanorod targets with a length of 60 *μ*m. Particle-in-cell simulations reveal that THz pulses are mostly generated by coherent transition radiation of laser produced hot electrons, which are efficiently enhanced with the use of nanorod targets. Good agreement is found between the simulation and experimental results.

Terahertz radiation is a powerful tool for imaging^1^ and probing various physical systems^2^, because of its unique nonionizing nature and transparency to many materials that are opaque to visible radiation^3^. It also has strong interactions with diverse materials, which allow THz radiation to probe many physical, chemical and biological systems^4^,^5^,^6^,^7^. Intense broadband THz pulses are also opening new scientific areas to explore, such as nonlinear optics in the THz domain^8^, as well as new technological opportunities, such as single-shot THz spectroscopy and imaging^9^. Stimulated by such applications, the generation of intense THz pulses has recently been the subject of great interest, and several table-top techniques have been studied in detail^10^,^11^. For example, optical rectification sources and THz generation from air plasma^12^,^13^ have been the subject of many extensive efforts to scale up both THz peak electric field and energy^10^. THz pulses with more than 100 MV/cm peak electric field have been demonstrated by difference-frequency mixing of two parametrically amplified pulses^14^. Electron accelerators can also be used to produce energetic THz pulses either (i) by bending a beam of relativistic electrons using a strong magnetic field or (ii) by sending them through a thin metal foil, but usually they are not available at modest laboratory scales^15^,^16^,^17^,^18^. Progress in research up to now has made it clear that many laser-based methods possess an upper limit in the maximum driving laser intensity^19^ that could be used, which is limiting the THz yield and hence their applications to several spectroscopic purposes^4^,^20^.

High-intensity ultrafast laser-plasma interaction can also be a potential source of intense ultra-broadband THz radiation^21^,^22^,^23^,^24^. Particle-in-cell (PIC) simulations have predicted that GV/cm THz fields can be generated by such interactions via different mechanisms such as laser wakefield excitation in underdense plasma^25^ and coherent transition radiation with solid targets^26^. Intense transition radiation in the THz frequency range has been observed when energetic electrons generated by high-intensity laser-solid interaction are ejected from the target^26^,^27^. THz pulses (0.1–133 THz) with pulse energy more than 700 μJ have been demonstrated from rear side of a foil target via a different mechanism; THz generation via target normal sheath acceleration^24^,^28^. Intense THz pulses via generation of coherent transition radiation in the rear side of size limited thin targets have also been demonstrated by Liao *et al*.^27^. Being generated via energetic electrons, transition radiation could reveal the characteristics of those electron bunches produced during the laser-solid interaction and hence is very important in high-intensity laser-plasma interaction^29^,^30^.

In this work, we first study transition radiation in the THz frequency range generated by relativistic intensity laser-plasma interaction using thick copper (Cu) targets. Then, we demonstrate that aligned Cu nanorod targets, which have been used to improve the efficiency of generating highly energetic photons and particles^31^,^32^,^33^,^34^,^35^,^36^,^37^, can also be used to significantly enhance the THz pulse energy through transition radiation. Single-shot electro-optic measurement shows the temporal behavior as well as the coherent nature of the THz pulses generated via transition radiation.

## Results

**Figure 1:**
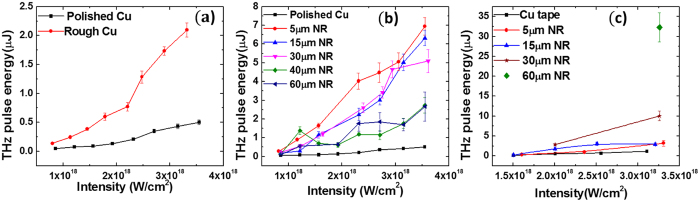
THz pulse energy for different targets as a function of laser intensity on target. (**a**) Comparison of THz pulse energy generated from thick polished and rough Cu solid targets in spectral range ≤20 THz; (**b**) Comparison of THz pulse energy generated from nanorod (NR) targets of different nanorod lengths (*h*) and diameter (D) of 200 nm in the spectral range ≤20 THz; (**c**) THz pulse energy generated from nanorod targets with different nanorod (NR) lengths, with high-pass window in spectral range from 20 THz to 200 THz.

In our experiments, intense, THz pulses are generated via high-intensity laser-solid interaction (for details about the experiments, please see Method). In Fig. 1(a), we compare the THz pulse energy for medium frequencies (≤20 THz, with UHMWPE window) detected by the pyroelectric detector from polished and rough Cu thick-solid targets. The presence of small random structures on the target surface could increase the THz pulse energy by about 4 times. In Fig. 1(b), we show the THz pulse energy emitted when high-intensity femtosecond laser irradiates the aligned Cu nanorod targets with a nanorod diameter (D) of 200 nm and lengths (*h*) of 5 μm, 15 μm, 30 μm, 40 μm, and compare them with the THz pulse energy emitted from an optically polished Cu target.

Compared with polished Cu targets, we observe a 13.8 times enhancement in the THz pulse energy for *h* = 5 *μ*m nanorod targets. However, we also find that the THz pulse energy decreases as nanorod length (*h*) increases further. This is in contrast to earlier studies on the X-ray generation, which showed enhancement in the X-ray yield with increasing nanorod length^34^. To investigate this difference, we repeat the experiment with a high-pass window that has lower transmission for lower frequencies (1% for ≤20 THz, ~10% transmission for ≥100 THz and ~60% for ≥130 THz). Radiation with frequencies higher than 200 THz is blocked by the HRFZ-Si filters. The results are shown in Fig. 1(c). In this measurement, the reference material is Cu tape, because the Cu nanorod targets were attached to the bulk Cu target by Cu tape, which has adhesives on both sides. For higher frequencies of electromagnetic radiation (20 THz to 200 THz), the pulse energy increases with the nanorod length (*h*), and the enhancement in the pulse energy reaches as high as 28 times for the case of the 60 *μ*m long Cu nanorod target, demonstrating a large pulse energy of about 32 *μ*J per pulse in 0.0873 Sr solid angle.

### Single-shot detection of THz pulses

**Figure 2:**
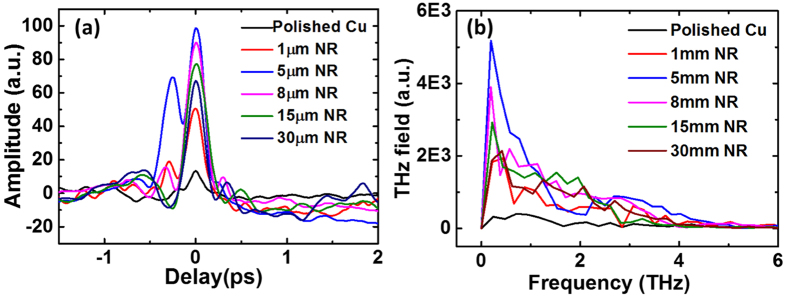
Comparison of THz pulses (**a**) in temporal domain (**b**) in frequency domain captured in single-shot experiment with polished and nanorod targets.

Temporal profiles of the THz pulses have been measured by using single-shot electro-optic measurements^38^ using a reflecting echelon mirror, a technique similar to the one developed by Minami *et al*.^39^. For details about experimental scheme, please see the section Methods. The THz field profile in the temporal domain as well as in the frequency domain obtained from polished and nanorod targets are shown in Fig. 2(a) and (b). The temporal profile of the THz pulses varies from shot to shot, and so in Fig. 2(a), we show the data that has the maximum THz peak field out of 20 successive shots. Likewise, THz energy measurement from nanorod targets, THz field measurement also shows maximum for 5*μ*m long Cu nanorod. A simple Gaussian fit provides a pulse width (FWHM) of 235±6 fs for the THz pulse obtained from 8 *μ*m nanorod target, which is expected by the ultra-broadband nature of the THz pulses.

### Numerical simulations

**Figure 3:**
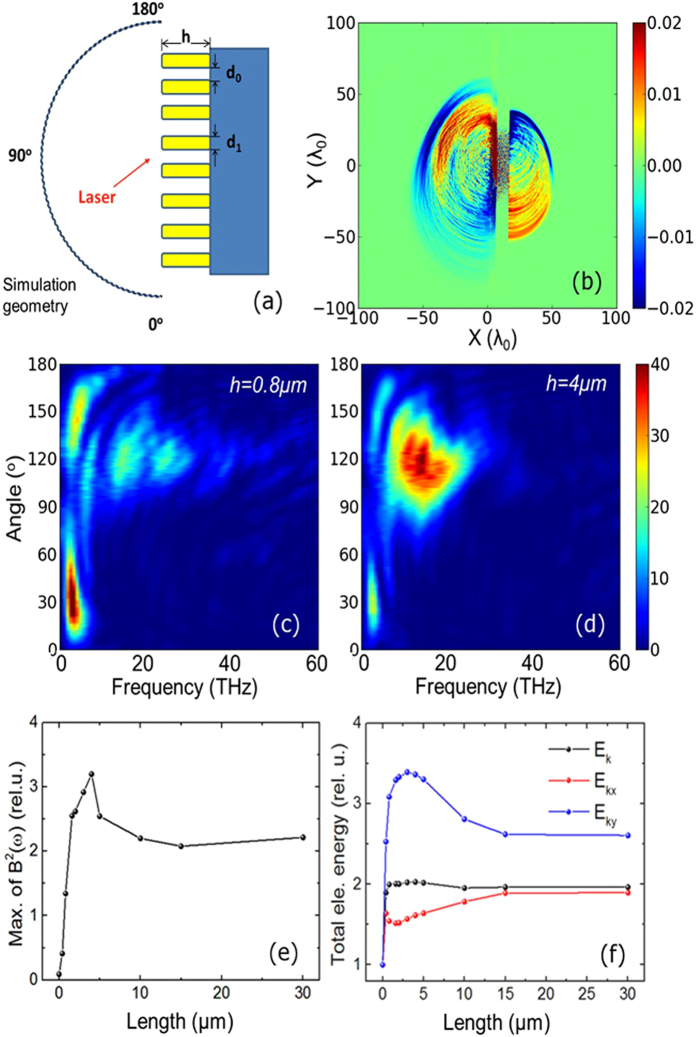
PIC simulation results: (**a**) Simulation geometry. (**b**) Snapshot of magnetic field Bz (averaged in a laser cycle) of the THz radiation. (**c**) and (**d**) Spectra of EM fields emitted from the target front with the nanorod length is 0.8 *μ*m and 4 *μ*m, respectively. (**e**) Intensity of THz radiation (maximum in THz range of the spectra) as a function of the nanorod length. Radiations are detected in the reflection direction (120°). (**f**) Total kinetic energy of hot electrons (*E*_*k*_ > 30 *keV*) as a function of the nanorod length. Energies are rescaled to that of planar target.

To understand our experimental results in detail, we have performed two dimensional particle-in-cell (PIC) simulations. The geometry of the simulation box is shown in Fig. 3(a). The simulation parameters are set close to those of the experiment. In the simulations, a *p*-polarized Gaussian beam with wavelength of 800 nm, pulse width of 40 fs and intensity of 3.5 × 10^18^ Wcm^−2^ is incident onto the target at an incidence angle of 30°. Since the prepulse contrast of the laser, which is used in the experiment is high, we have not included preplasma in the simulations. An 8 *μ*m thick copper target is used, which is covered with Cu nanorod arrays with 200 nm diameter (D) and spacing (*d*_0_) of 200 nm. Figure 3(b) is a snapshot of the spatial distribution of the magnetic field, which is time-averaged over a laser period in order to filter out the high frequency components. Both backward and forward radiations are emitted from the target front and back sides, respectively, which can be attributed to coherent transition radiation generated by hot electrons produced in the laser-plasma interaction^26^,^40^. For polished planar targets, the THz radiation is the weakest around the specular reflection direction and strongest along the target surface direction, typical for transition radiations. The simulation shows that the nanorods have enhanced the backward THz radiation and have changed its emission direction to the specular reflection direction, significantly from that predicted for a planar target. These are more obvious from the spectra of the electromagnetic fields, which is detected 100 *μ*m away from the laser irradiated spot in the target front, as shown in Fig. 3(c) and (d).

To check the effect of the nanorod length on the THz radiation, we have varied the nanorod length from 0 to 30 *μ*m, which shows the presence of optimized nanorod length for THz radiation. When the nanorod length increases from 0 to 4 *μ*m, the radiation intensity decreases along the target surface (near 0° or 180°) while it increases in the specular reflection direction (120°). As a whole, it becomes dominant on the specular reflection direction. Figure 3(e) shows that the intensity of the backward THz radiation in the specular reflection direction varies with the nanorod length, where the intensities are normalized to the maximum THz emission from polished targets. As the length increases, the radiation intensity increases until *h* = 4 *μ*m, then it decreases slightly for *h* > 4 *μ*m, which reaches a saturation for *h* > 10 *μ*m. Because the emitted THz radiation is coherent transition radiation generated by the hot electrons, the radiation is the strongest when the absorption rate of the laser energy by electrons is the highest. The corresponding optimal nanorod length, at which the highest number of hot electrons is produced, is determined by the nanorod parameters *d*_0_ and *d*_1_^36^. The simulations show enormous increase in both the number and total energy of hot electrons for the case of nanorod targets, when compared with polished targets. The variation in the total kinetic energy of hot electrons as a function of the nanorod length (*h*) is shown in Fig. 3(f). The nanorod array enhances the electron energy in both the *x (E*_*kx*_) and *y (E*_*ky*_) directions, but much more significantly in the *y* (vertical) direction. We observe that *E*_*ky*_ peaks at around *h* = 3 *μ*m, although the energy in the horizontal *x*-direction *E*_*kx*_ is not the maximum. The electron energy in the *y*-direction (*E*_*ky*_) contributes more to THz emission in specular reflection direction (120°) than the electron energy in the *x*-direction (*E*_*kx*_). This is consistent with the theory of coherent transition radiation that the radiation is mostly emitted in a large angle or nearly perpendicular to the moving direction of the electron^26^,^40^,^41^ when the electrons are of moderate energy. The average kinetic energy of hot electrons is normally at the order of 100 keV under the laser conditions. The maximum *E*_*ky*_ is about 3.4 times higher than that from polished targets. The tendency of the THz intensities agrees very well with the behavior of the total energy of hot electrons in the vertical direction.

## Conclusions

We have demonstrated that the THz pulse energy can be increased by using aligned Cu nanorod array targets, with a maximum enhancement of up to 13.8 times for ≤20 THz and 28 times in the spectral range between 20 THz and 200 THz. It is shown that there is an optimal nanorod length for the most efficient THz radiation. The temporal profiles of the THz pulses are also obtained by the use of a single-shot electro-optic measurement. PIC simulations reveal the mechanism behind the intense THz pulse generation by this technique, which we attribute to coherent transition radiation at THz frequencies by the energetic electrons produced by the laser plasma interactions. It also reveals that the THz radiation with nanorod array targets is distributed mainly in the specular reflection direction, which is different from that with a planar target.

The THz pulses, especially for low frequency THz radiation, are emitted in a broad angle, which is also seen in the simulation results. However, we only collect THz signal over a small solid angle of 0.0873 Sr, and thus we obviously do not collect all of the THz energy generated by the interaction. To estimate the total THz pulse energy generated by the interaction, we consider that the THz pulses are emitted in a 2*π* solid angle^24^,^42^ on the target front. By correcting for the small solid angle of detection, we estimate that several hundred micro-joule THz energy is generated at a frequency range (≤20 THz). Most of this THz pulse energy can easily be collected by using an ellipsoidal mirror^24^, which opens a roadway towards millijoule class THz sources on tabletop.

## Methods

**Figure 4:**
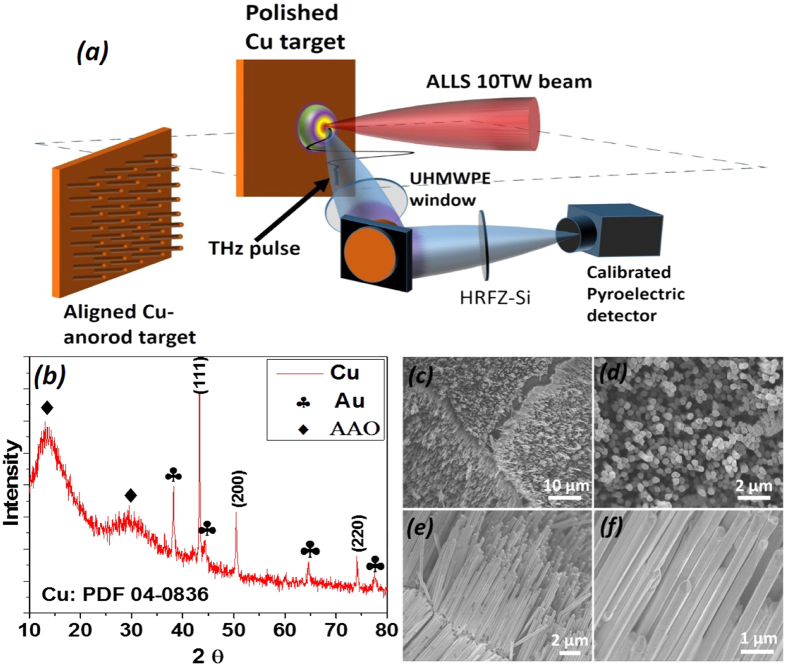
(**a**) Schematic diagram of the experimental setup. (**b**) X-ray diffraction (XRD) spectrum of Cu nanorods embedded in AAO template. (**c**), (**d**), (**e**) & (**f**) SEM images of Cu nanorod arrays at different magnifications: (**c,d**) top view; (**e,f**) cross-sectional view.

Figure 4(a) shows a schematic diagram of the experimental setup. We use 10 TW femtosecond laser pulses with high contrast (nanosecond contrast ~10^−7^) from the10 Hz beam line of the Advanced Laser Light Source (ALLS) facility at INRS-EMT. This laser, with incidence angle of 45 and *p*-polarization, is focused on to a Cu target (size: 5 cm × 5 cm × 3 cm) using an f/3 off-axis parabolic mirror, to a circular spot of 20 *μ*m in diameter. The target is mounted on an automated XYZ translation stage inside a vacuum chamber. The 10 Hz beam line can deliver up to 240 mJ maximum pulse energy with 40 fs pulse duration after compression, with a central wavelength of 800 nm, which when focused results in a peak intensity of about 3.5 × 10^18^ Wcm^−2^. Ultra-broadband THz and infrared (IR) pulses (via the generation of coherent transition radiation) are generated as a result of laser-plasma interaction. THz pulses are then collimated by using a thick gold plated off-axis parabolic mirror, and then guided out of the vacuum chamber through a THz window made from ultrahigh molecular weight polyethylene (UHMWPE), which only transmits radiation up to 20 THz while blocking higher frequency electromagnetic radiation. The generated THz pulses are then refocused on to a calibrated pyroelectric detector (Gentec-EO, THZ5I-BL-BNC) using another off-axis parabolic mirror to measure the THz pulse energy. To remove any residual of the 800 nm driving laser, we have further used two high-resistivity float-zone silicon (HRFZ-Si) filters that have flat transmission up to the cut-off at around 1.5 *μm*. We take an average over 15 shots to improve the statistical error. The THz pulse energy from the optically polished target is compared with that from the aligned Cu nanorod array targets. The THz pulse energy is collected over a relatively small solid angle of 0.0873 Sr.

### Nanorod target fabrication

The commercially available porous anodic aluminum oxide (AAO) membrane (pore size: 200 nm, membrane thickness: 60 *μ*m, *Whatman*^®^ Anodisc) was used as a template for the electrochemical deposition of Cu nanorod arrays^43^. A gold layer (300 nm) was sputtered on one side of the through-hole AAO template, serving as the working electrode in a conventional three-electrode cell for Cu electrochemical deposition, with graphite carbon and saturated calomel electrode (SCE) electrode as the counter and reference electrode, respectively. The electrolyte was 0.2 M CuSO_4_·5H_2_O + 0.1 M H_3_BO_3_ for Cu deposition. Experiments were carried out using Potentiostat (Autolab) with the constant potential of −1.20 V (vs. SCE) at room temperature. The length of the Cu nanorods can be controlled between 0 and 60 *μm* by adjusting the deposition time.

The crystallographic studies of Cu nanorods embedded in AAO were carried out by X-ray spectroscopy (XRD, Bruker D8 Advanced Diffractometer, Cu *Kα* radiation). The XRD spectrum of the Cu nanorods, shown in Fig. 4(b), fits the standard XRD pattern very well. Three reflection peaks attributed to (111), (200), (220) are evidently noticeable, and can be completely indexed to the Cu face-centered cubic crystal structure (JCPDS 04-0836)^44^. There are no impurities except for the broad peaks belonging to the amorphous AAO template and the corresponding peaks of the sputtered Au electrode. For the SEM characterization, the as-prepared Cu nanorods embedded in AAO template were first immersed in the NaOH solution to dissolve the alumina membrane, and then the nanorods were washed thoroughly with distilled water and ethyl alcohol several times. Figure 4(c), (d), (e) and (f) show the SEM images of the Cu nanorods (length *h* = 15 *μm*) at different magnifications. It is clearly depicted from the SEM micrograph that a large quantity of well-aligned, dense, homogeneous in diameter, and parallel to each other Cu nanorods have been successfully fabricated by this technique.

### Single-shot electro-optic measurement

**Figure 5:**
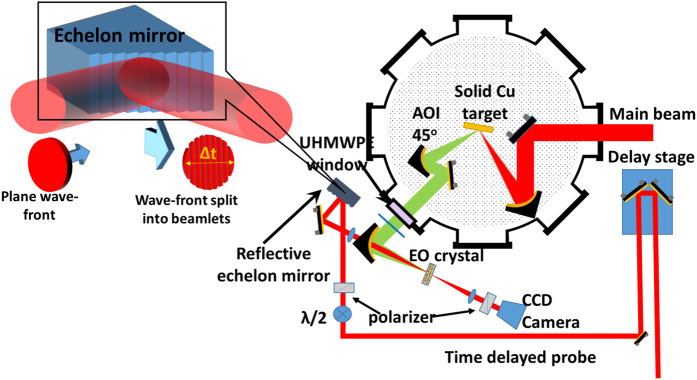
Schematic of the experimental setup for single-shot electro-optic measurement of THz pulses. The left panel shows the working mechanism of reflective echelon mirror.

The single-shot electro-optic measurement of THz pulses is setup by carefully encoding the temporal information in the spatial direction^38^,^39^,^45^,^46^. The THz pulses generated by this technique and described before are then refocused on an electro-optic crystal by using another off-axis parabolic mirror, which has a hole at the center. A 1mm thick Zinc-Telluride (ZnTe) crystal has been used as the electro-optic crystal in our case, which has spectral response up to 3 THz. The spectral sensitivity of the THz detection system is limited to 3 THz by the ZnTe crystal. A part of the main beam (~1mJ in energy), which can be time delayed by a 5 cm delay stage in the path, as shown in the Fig. 5, has been used as a probe for the electro-optic sampling. The probe energy is then reduced further below the damage threshold of the electro-optic crystal, which is placed at the focus, by a thin neutral density filter. Before interacting with the THz pulse on the ZnTe crystal, the probe beam passes through a half-wave (λ/2) plate and a thin polarizer and reflects from a nickel echelon mirror. The half-wave (λ/2) plate is used to overlap the plane of polarization between the probe beam and the generated THz beam. The thin polarizer is used to increase the polarization contrast of the input probe. The delay line in the conventional electro-optic measurement of THz pulses is replaced by the echelon mirror. The echelon mirror^46^ consists of a number of optically polished reflecting steps of equal dimension distributed horizontally, as shown in the left panel of Fig. 5. In our case, the echelon mirror with dimension of 10 mm × 10 mm is used, which is made with polished nickel with 500 steps of height H = 5 *μ*m and width W = 20 *μ*m and is placed just before the interaction with the THz pulses.

A plane wave front, after reflection from the echelon mirror, splits into several beamlets with a delay between them, thus encoding the temporal information in the spatial direction. The beamlet size and the delay between beamlets is determined by the step size of the echelon mirror. Any space resolved detector such as CCD/CMOS camera with sufficient pixel resolution can be used to extract temporal information.

After reflection from the echelon mirror, the probe beam is then focused through the central hole of the off-axis parabolic mirror and finally collinearly overlaps with the THz beam on the ZnTe crystal. After being collimated by another lens with the same focal length, the spatial profile of the probe beam is captured on a CCD camera (Point Grey: FL2-14S3M-C), which is triggered by the laser. A Glan polarizer has been placed just before the CCD camera, which works as an analyzer to capture any change in the polarization state of the probe pulse due to the electro-optic effect during interaction with the THz pulses. Initially, the temporal overlap of the 800 nm probe pulse with the attenuated 800 nm pump pulse has been performed by looking at the interference fringe at the CCD camera and with the help of a delay line incorporated in the beam path, as shown in Fig. 5. After that, THz window made from UHMWPE and HRFZ-Si filters are used in main beam path to block the IR beam and only THz beam (≤20THz) is allowed to pass. The delay stage in the probe path is then readjusted for proper positioning of the THz peak in the THz trace in the CCD image by looking at the image from the CCD camera.

## Additional Information

**How to cite this article**: Mondal, S. *et al*. Aligned copper nanorod arrays for highly efficient generation of intense ultra-broadband THz pulses. *Sci. Rep.* **7**, 40058; doi: 10.1038/srep40058 (2017).

**Publisher's note:** Springer Nature remains neutral with regard to jurisdictional claims in published maps and institutional affiliations.
